# Transformations of Organic Molecules with F-TEDA-BF_4_ in Ionic Liquid Media

**DOI:** 10.3390/molecules14072394

**Published:** 2009-07-06

**Authors:** Jasminka Pavlinac, Marko Zupan, Stojan Stavber

**Affiliations:** 1 Department of Physical and Organic Chemistry, ‘Jožef Stefan’ Institute, Jamova 39, 1000 Ljubljana, Slovenia; E-mail: jasminka.pavlinac@ijs.si (J.P.); 2 Faculty for Chemistry and Chemical Technology, University of Ljubljana, Aškerčeva 5, 1000 Ljubljana, Slovenia; E-mail: marko.zupan@fkkt.uni-lj.si (M.Z.)

**Keywords:** F-TEDA-BF_4_, fluorination, ionic liquids, green chemistry

## Abstract

The transformations of organic molecules with F-TEDA-BF_4_ (**1**) were investigated in the hydrophilic ionic liquid (IL) 1-butyl-3-methyl-imidazolium tetrafluoroborate ([bmim][BF_4_], **2**) and the hydrophobic IL 1-butyl-3-methyl-imidazolium hexafluorophosphate ([bmim][PF_6_], **3**). The range of substrates included alkyl substituted phenols **4a-c**, **9**, **13**, 1,1-diphenylethene (**15**), alkyl aryl ketones **19**-**22**, aldehydes **23**-**25** and methoxy-substituted benzene derivatives **26**-**30**. The evaluation of the outcome of reactions performed in IL media in comparison to those of the corresponding reactions in conventional organic solvents revealed that the transformations in IL are less efficient and selective. The effect of the presence of a nucleophile (MeOH, H_2_O, MeCN) on the course of reaction was also studied.

## 1. Introduction

The last decade has witnessed a rapidly growing interest in various aspects of ionic liquids (ILs), with great emphasis being placed on their potential utility in chemical synthesis as recyclable solvents, reagents and catalysts [1,2]. Prime features that have made ILs so attractive include their low vapour pressure, high thermal and chemical stability, wide liquid-state temperature range, large electrochemical window, favourable solvation behavior and their potential for recyclability, all of which lead to their recognition as ‘greener’ alternative media to volatile and often toxic organic solvents [3,4,5,6]. Additionally, their highly diverse chemical and physical properties resulting from the infinite possibilities for combining cations and anions to form ILs are also considered promising advantages for their various applications, e.g. in synthetic transformations, catalysis, electrochemistry, spectroscopy, extraction and separation processes [7,8,9,10]. However, a full understanding of their physical and chemical behavior is still lacking and data on their environmental effects have only just started to appear [11,12,13,14], so one should be extremely careful before claiming that a certain transformation is advantageous and environmentally friendlier if performed in ILs rather than in conventional organic solvents.

Owing to its unique properties (e.g. small size, high electronegativity, low polarizability, strong C-F bond), the incorporation of fluorine into organic molecules may bring about a dramatic change in their physical, chemical and biological properties [15]. An increased demand for fluorinated compounds for diverse applications, such as pharmaceuticals, agrochemicals, solvents, liquid crystals, dyestuffs, polymers and novel materials, has been notable in recent decades and consequently, fluorination of organic molecules has become a very important target in various sectors [16,17]. Although substantial effort has been devoted to the development of methods for the selective introduction of fluorine into target molecules, mild, selective and environmentally more acceptable fluorination procedures still continue to remain a significant challenge to organofluorine chemists. Due to the lack of appropriate reagents at the beginning of the 20^th^ century, fluorination of organic compounds represented an extremely demanding area of organic chemistry. Reactions employing molecular fluorine are, due to the weak F-F bond and strong C-F bond formed, characterized by high exothermicity, and thus its handling requires working at very low temperatures and pressure, using special laboratory equipment and exceptional safety precautions. Further developments included reagents such as XeF_2_ and O-F reagents, while a breakthrough was achieved with the introduction of N-F reagents. The latter have been recognized as mild and efficient electrophilic fluorinating reagents, whose handling does not require special safety measures. Among them 1-chloromethyl-4-fluoro-1,4-diazoniabicyclo[2.2.2]octane bis(tetrafluoroborate), F-TEDA-BF_4_ (**1**), known under the commercial name of Selectfluor™, has been highlighted as an efficient and selective fluorinating reagent for a wide variety of organic compounds, in addition to possessing oxidative properties and as such being utilized also as a mediator or a catalyst for other functionalizations [18,19,20,21]. This reagent has been extensively studied for various transformations in conventional organic solvents, while in ILs it has been investigated only for a limited series of compounds [22,23,24,25,26].

**Scheme 1:**
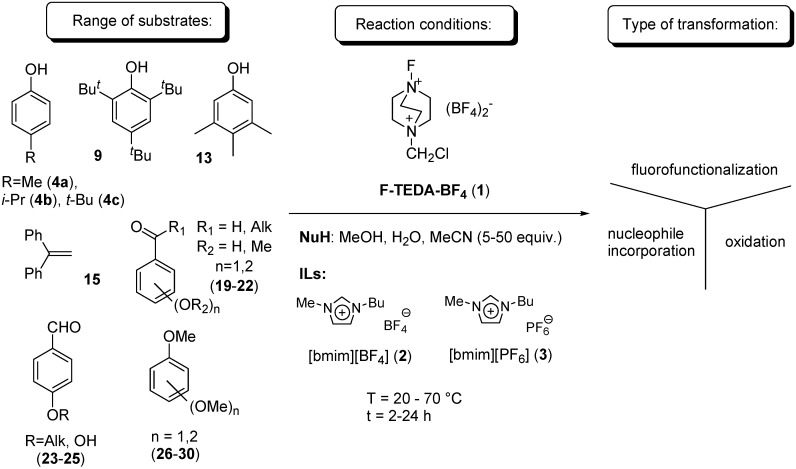
Effect of reaction conditions on the course of transformation of organic molecules with F-TEDA-BF_4_ in the presence of a nucleophile.

The present study aimed to investigate transformations of alkyl substituted phenols **4a**-**c**, **9**, **13**, 1,1-diphenylethene (**15**) as a model alkene molecule, methoxy substituted alkyl aryl ketones **19**-**22**, aldehydes **23**-**25** and methoxy-substituted aromatic substrates **26**-**30**, and to compare the results obtained in IL media with the outcomes of the corresponding reactions in conventional organic solvents. Especially for alkyl substituted phenols [27,28,29,30], alkenes [31,32,33] and aryl alkyl ketones [34], it has been previously established that the reaction conditions may play a significant role on the course of the transformation, while many examples display the dramatic effect of ionic liquids on the selectivity and efficiency of reaction. This study was performed using the hydrophilic IL 1-butyl-3-methyl-imidazolium tetrafluoroborate ([bmim][BF_4_], **2**) and hydrophobic 1-butyl-3-methyl-imidazolium hexafluorophosphate ([bmim][PF_6_], **3**). The effect of a nucleophile on the course of reaction was additionally investigated (Scheme 1).

## 2. Results and Discussion

First of all we performed a blank experiment by stirring solutions of 0.24 mmol of F-TEDA-BF_4_ in 1 mL of ILs [bmim][BF_4_] or [bmim][PF_6_] at 70^o^C for 24 h and established that no reaction between the fluorinating reagent and either IL occurred. The fluorinating reagent F-TEDA-BF_4_ remained active, while no transformation of [bmim][BF_4_] or [bmim][PF_6_] could be observed from NMR spectra.

**Scheme 2:**
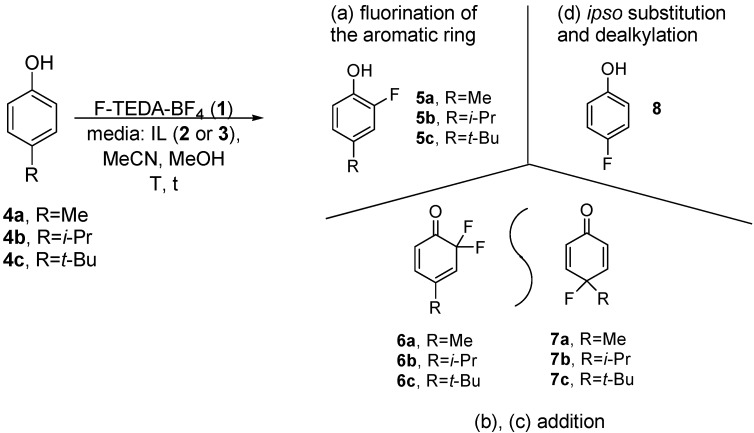
Transformation of 4-alkyl substituted phenols with F-TEDA-BF_4_.

The study of fluoro transformations of alkyl substituted phenols with F-TEDA-BF_4_ (**1**) in the ILs [bmim][BF_4_] and [bmim][PF_6_] revealed that the properties of the IL have a considerable effect on the course of transformation, as well as on its efficiency. Likewise, in conventional organic solvents, depending upon the substrate structure and reaction conditions, the following products may be present in the reaction mixture when employing IL media: (a) 2-fluoro-4-alkylphenols **5**, as a consequence of electrophilic substitution at the *ortho* position with respect to the phenolic group; (b) difluorocyclohexadienone (**6**) after further fluorination of *ortho*-fluoro substituted phenolic product **5**; (c) 4-alkyl-4-fluoro-cyclohexa-2,5-dienone (**7**) after the addition-elimination process following an attack at the *para* position with respect to the phenolic group, and (d) 4-fluorophenol (**8**) as a result of *ipso* substitution after dealkylation at the *para* position (Scheme 2). However, their distribution in ILs (Table 1) differs from the corresponding product distribution obtained in MeCN or in MeOH [27]. 

A typical experiment was performed in the following way: 0.24 mmol of F-TEDA-BF_4_ was vigorously stirred in 1 mL IL until the reagent was completely dissolved. Then, 0.2 mmol of substrate was added and the reaction mixture was continued to be stirred at temperature similar to the corresponding reaction in an organic solvent. After reaction ceased, the reaction mixture was extracted with *t*-BuOMe, the organic phase washed with water and after drying, filtered, concentrated *in vacuo* and the crude reaction mixture analysed by TLC, ^1^H- and ^19^F-NMR.

**Figure 1:**
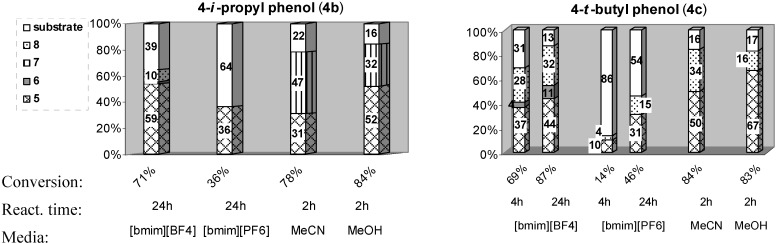
Comparison of product distribution obtained in reaction of 4-*i*-propyl phenol (**4b**) or 4-*t*-butyl phenol (**4c**) with F-TEDA-BF_4_ in ILs and organic solvents^a^.

^a^* Reaction conditions*: in [bmim][BF_4_] and [bmim][PF_6_]: 0.2 mmol substrate, 0.24 mmol F-TEDA-BF_4_, 1 mL IL, 70 °C; in organic solvent [27]: 1 mmol substrate, 1 mmol F-TEDA-BF_4_, 10 mL solvent, reflux.

In the case of 4-methylphenol (**4a**), the transformation was less efficient in both studied ILs than in MeCN or MeOH [27], since the formation of a complex reaction mixture or low transformation of starting material was observed (entries 1-3; Table 1). Compared to **4a**, 4-*i*-propyl phenol (**4b**) and 4-*t*-butyl phenol (**4c**) were transformed more efficiently in IL media (Table 1, entries 4-6), but still less efficiently compared to the reaction carried out in an organic solvent [27]. In a 24 h reaction between **4b** and F-TEDA-BF_4_ carried out at 70 °C in [bmim][BF_4_], the substrate was 71% converted into a mixture of fluorinated products, with 2-fluoro-4-*i*-propyl phenol (**5b**) as the major product and 2,2-difluoro-4-*i*-propyl-3,5-cyclohexadienone (**6b**) and 4-fluorophenol (**8**) as minor ones. Under comparable reaction conditions in the hydrophilic IL [bmim][PF_6_], only the *ortho*-fluoro substituted product **5b** was formed, however with a modest conversion (36%). The transformation proceeded more efficiently in hydrophilic [bmim][BF_4_] than in [bmim][PF_6_], as was the case of 4-*t*-butylphenol (**4c**) (Table 1, entries 5, 6). In the reaction between **4c** and F-TEDA-BF_4_ carried out at 70 °C for 4 h in [bmim][BF_4_], 69% of the substrate was converted into a mixture of 3 products, namely 2-fluoro-4-*t*-butylphenol (**5c**, 37%), 4-fluorophenol (**8**, 28%) and 2,2-difluoro-4-*t*-butyl-3,5-cyclohexadienone (**6c**, 4%), while 31% of the substrate remained unchanged (Table 1, entry 5). Under the same reaction conditions applied to the reaction between **4c** and F-TEDA-BF_4_ in [bmim][PF_6_], 86% of the substrate remained unreacted, the rest corresponding to *ortho*-fluoro substituted product **5c** (10%) and 4-fluorophenol (**8**, 4%). Prolonging the reaction time to 24 h increased to the efficiency of the transformation (entry 6). In [bmim][PF_6_] 46% of **4c** was converted into a 2:1 mixture of 2-fluoro-4-*t*-butylphenol (**5c**) and 4-fluorophenol (**8**) (Table 1, entry 6). Comparing the outcome of reactions for these substrates with F-TEDA-BF_4_ performed in organic solvents (MeCN, MeOH) or in IL media ([bmim][BF_4_], [bmim][PF_6_]), a more efficient and faster reaction was observed to take place in organic solvents than in ILs, while the distribution of products were also found to be considerably different (Figure 1).

**Table 1:** Transformations of 4-alkyl substituted phenols with F-TEDA-BF_4_ in ILs. 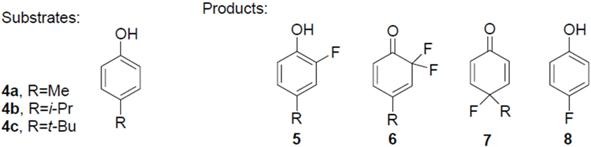

Entry	R	IL^a^	[bmim][BF_4_]		[bmim][PF_6_]	
		T, t	Conv.^b^	Product distribution (5 : 6 : 7 : 8)	Conv.^b^	Product distribution (5 : 6 : 7 : 8)
1	Me ( **4a**)	70 °C, 4 h	complex reaction mixture		complex reaction mixture	
2		22 °C, 22 h	10%	6 : 0 : 4 : 0	0^c^	
3		50 °C, 22 h			20%	100 : 0 : 0 : 0
4	*i*-Pr (**4b**)	70 °C, 24 h	71%	59 : 2 : 0 : 10	36%	100 : 0 : 0 : 0^d^
5	*t*-Bu (**4c**)	70 °C, 4 h	69%	37 : 4 : 0 : 28	14%	10 : 0 : 0 : 4
6		70 °C, 24 h	87%	44 : 11 : 0 : 32	46%	31 : 0 : 0 : 15^e^

^a^ Ionic liquid; ^b^ Conversion of substrate determined by ^1^H- and ^19^F-NMR; ^c^ Unreacted substrate; ^d^ The presence of **6b** and **8** in traces; ^e^ The presence of **6c** in traces.

**Scheme 3:**
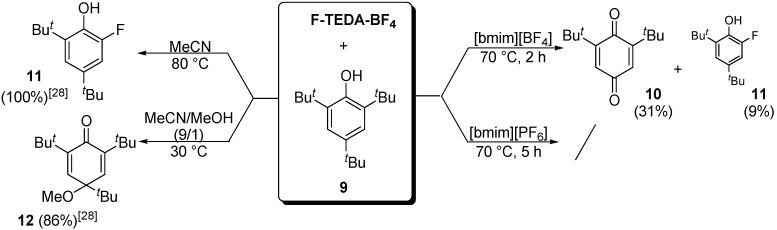
Effect of reaction conditions on the reaction of 2,4,6-tri-t-butyl phenol (**9**) with F-TEDA-BF_4_.

In the case of a sterically hindered alcohol, namely 2,4,6-tri-*t*-butylphenol (**9**, Scheme 3), the reaction with F-TEDA-BF_4_ in [bmim][PF_6_] did not proceed at all in 5 h either at room temperature or at 70 °C. After 2 h reaction carried out at 70 °C in [bmim][BF_4_], 60% of the substrate remained unreacted, while 31% of oxidized quinone product **10** was formed along with 9% 2-fluoro-4,6-di-*t*-butylphenol (**11**). This result is different from the outcome of the reaction performed in MeCN or MeOH, where as earlier established, the polarity of the solvent and reaction temperature greatly affected the course of reaction [28,29]. In MeCN, the substrate **9 **was quantitatively transformed into the fluoro-dealkylated product **11** at 80 °C, while a mixture of MeCN/MeOH (9/1 ratio) at 30 °C proved to be suitable for the quantitative formation of 4-methoxy-2,4,6-tri-*t*-butyl-cyclohexa-2,5-dienone (**12**).

**Scheme 4:**
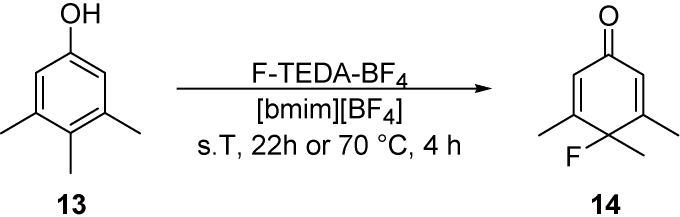
Transformation of 3,4,5-trimethylphenol (**13**) with F-TEDA-BF_4_ in the hydrophilic IL [bmim][BF_4_].

3,4,5-Trimethylphenol (**13**) in [bmim][BF_4_] was quantitatively converted into 4-fluoro-3,4,5-trimethyl-2,5-cyclohexadienone (**14**) when it was reacted with F-TEDA-BF_4_ for 22 h at room temperature or for 4 h at 70 °C (Scheme 4). On the contrary, the reaction carried out in [bmim][PF_6_] at room temperature was inefficient, while at elevated temperature a polymeric material was formed. Likewise for the previously discussed substrates, MeCN seems to be an advantageous solvent for this transformation, since the product **14** was obtained efficiently in a reaction performed for 4.5 hours at room temperature [30].

Furthermore, we investigated the effect of a nucleophile on the transformation of 4-*t*-butylphenol (**4c**) and the sterically more hindered 2,4,6-tri-*t*-butylphenol (**9**) in reaction with F-TEDA-BF_4_ performed in an IL. After F-TEDA-BF_4_ was completely dissolved in the IL, the substrate (**4c** or **9**) was added to the reaction mixture, followed by the addition of approximately 5 molar equivalents of MeOH or MeCN and the reaction mixture continued to be stirred at an optimized reaction temperature. In the case of 4-*t*-butylphenol (**4c**), the presence of MeOH or MeCN in [bmim][BF_4_] did not have a substantial effect on the course of the reaction as transformation of the substrate, as well as the distribution of products **5c**, **8** and **6c**, were comparable to the results of reactions performed in the absence of the nucleophile. In the case of 2,4,6-tri-*t*-butylphenol (**9**), the substrate still prevailed (50-66%) in the reaction mixture when the reaction with F-TEDA-BF_4_ was performed in [bmim][BF_4_] and in the presence of 4-5 equivalents of a nucleophile (MeCN or MeOH), where 2,6-di-*t*-butyl-*p*-benzoquinone (**10**, 26-45%) was formed as the major product. In addition to the quinonic product **10**, the presence of a minor amount of dealkylated methoxy substituted di-*t*-butylphenols (2-methoxy-4,6-di-*t*-butylphenol and 4-methoxy-2,6-di-*t*-butylphenol) could be noticed from the NMR spectra if the reaction was carried out at 70 °C; however, these products were present in small quantities. Since in the mixture of MeCN/MeOH (9/1) the substrate **9** was converted with high yield into 4-methoxy-2,4,6-tri-*t*-butyl-cyclohexa-2,5-dienone (**12**) at 30 °C in few hours, one can conclude that an organic solvent is more advantageous for these transformations than the use of ILs (Scheme 3).

**Scheme 5:**
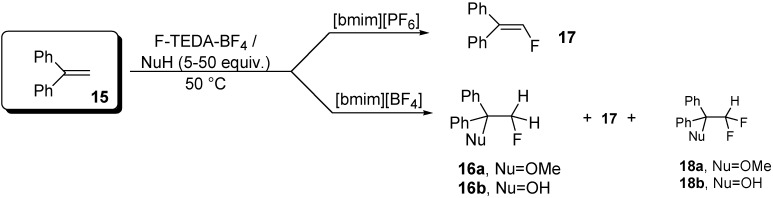
The selectivity of fluorotransformation of 1,1-diphenyl ethane (**15**) in reaction with F-TEDA-BF_4_ in an IL.

The effect of the presence of a nucleophile in IL media was further investigated for the fluorofunctionalization of 1,1-diphenylethene (**15**) as a model alkene substrate, with F-TEDA-BF_4_. The reaction between the substrate **15** and F-TEDA-BF_4_ performed in [bmim][PF_6_] at room temperature for 24 h did not provide any product, while under similar reaction conditions applied in [bmim][BF_4_] a modest transformation occurred, yielding 1,1-diphenyl-1-hydroxy-2-fluoroethane (**16b**, 25%) and 1,1-diphenyl-2-fluoroethene (**17**, 17%) (Table 2). Raising the temperature to 50 °C resulted in the almost complete conversion of the substrate in the hydrophilic IL [bmim][BF_4_] (Table 2, entry 7), while in [bmim][PF_6_] the conversion yield of the reaction performed at 50 °C and in the absence of a nucleophile was still modest (Table 2, entry 2). The experimental data indicate a difference in the selectivity of fluorotransformation obtained in an IL with [BF_4_] as the anionic part from the outcome of the reaction carried out in [bmim][PF_6_] (Scheme 5). In the latter, which forms a two-phase system with water, the substrate was converted to addition-elimination product 1,1-diphenyl-2-fluoroethene (**17**) with high selectivity, regardless of the presence of a nucleophile (MeOH, H_2_O, MeCN) (Table 2, entries 3-5). On the other hand, the addition product, namely vicinal hydroxy- or methoxyfluoroalkane **16b**, **16a**, prevailed in the reaction mixture when [bmim][BF_4_] was used as the reaction medium (Table 2, entries 8, 9).

**Table 2:** Effect of reaction conditions on fluorotransformation of 1,1-diphenyl ethene (**15**) with F-TEDA-BF_4_ in ILs^a^. 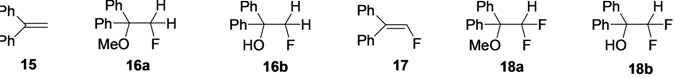

Entry	IL^b^	NuH^c^	T [°C]	Product distribution^d^					
				15	16a	16b	17	18a	18b
1	[bmim][PF_6_]	/	22	100%	/	/	/	/	/
2		/	50	64%	/	/	36%	/	/
3		MeOH	50	7%	/	/	60%	22%	12%
4		H_2_O	50	13%	/	/	78%	/	9%
5		MeCN	50	30%	/	/	70%	/	/
6	[bmim][BF_4_]	/	22	58%	/	25%	17%	/	/
7		/	50	2%	/	65%	14%	/	19%
8		MeOH	50	/	76%	6%	1%	17%	/
9		H_2_O	50	3%	/	76%	/	/	21%

^a^* Reaction conditions*: 0.2 mmol substrate, 0.24 mmol F-TEDA-BF_4_, 1 mL IL, 24h; ^b^ Ionic liquid; ^c^ nucleophile (5 molar equivalents); ^d^ The distribution of products determined from ^1^H- and ^19^F- NMR spectra of the crude reaction mixture.

**Figure 2:**
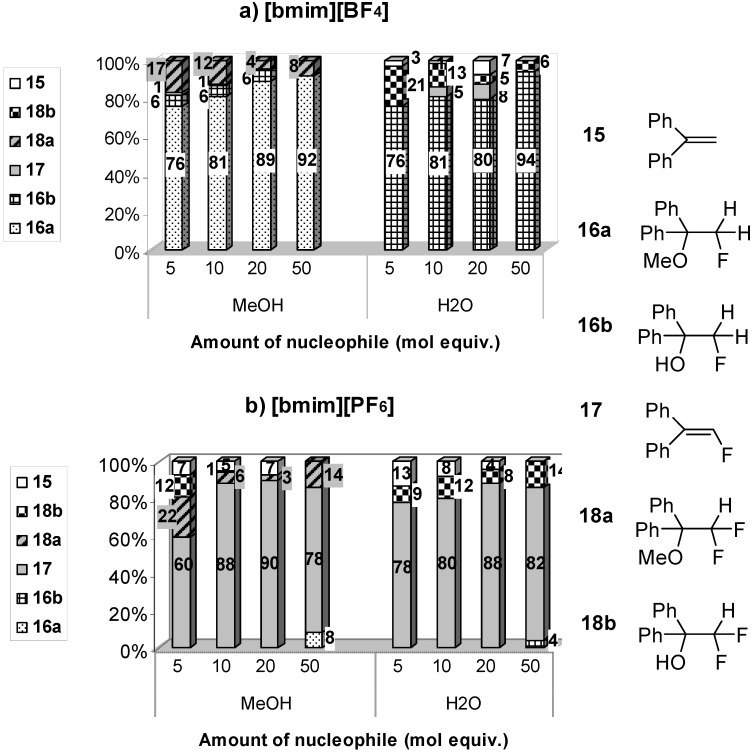
Effect of the amount of nucleophile on the product distribution for fluorination of 1,1-diphenyl ethane (**15**) with F-TEDA-BF_4_ in [bmim][BF_4_] and [bmim][PF_6_]^a,b^.

^a^* Reaction conditions*: 0.2 mmol substrate (**15**), 0.24 mmol F-TEDA-BF_4_, nucleophile (MeOH or H_2_O; 5, 10, 20 or 50 molar equivalents), 1 mL IL ([bmim][BF_4_] or [bmim][PF_6_]), 50 °C, 24 h; ^b^ The distribution of products determined from ^1^H- and ^19^F-NMR spectra of the crude reaction mixture.

Additionally, we investigated the effect of various amounts of a nucleophile (MeOH or H_2_O; 5, 10, 20 and 50 molar equivalents) on the fluorination of 1,1-diphenylethene (**15**) with F-TEDA-BF_4_ in both studied ILs (Figure 2). We found that the presence of 10-20 molar equivalents of MeOH or H_2_O in [bmim][PF_6_] considerably enhanced the efficiency of transformation of **15** into 1,1-diphenyl-2-fluoroethene (**17**). Furthermore, 1,1-diphenylethene (**15**) with F-TEDA-BF_4_ and in the presence of 5-50 equivalents of MeOH was completely converted after 24 h at 50 °C if the reaction was carried out in [bmim][BF_4_], thus highlighting the improved efficiency of the transformation in the presence of a nucleophile in this hydrophilic IL. In these cases, the major product corresponded to 1,1-diphenyl-1-methoxy-2-fluoroethane (**16a**), while 1,1-diphenyl-2-fluoroethene (**17**), 1,1-diphenyl-1-hydroxy-2-fluoroethane (**16b**) and 1,1-diphenyl-1-methoxy-2,2-difluoroethane (**18a**) could be detected in minor amounts from the NMR spectra as well. In cases where H_2_O was used as the source of the external nucleophile in [bmim][BF_4_], 1,1-diphenyl-1-hydroxy-2-fluoroethane (**16b**) formed as the major product, while the addition-elimination product **17** and 1,1-diphenyl-1-hydroxy-2,2-difluoroethane (**18b**) were present as minor products. *Vicinal* methoxy- or hydroxy-fluoroalkane (**16a** or **16b**) were produced most efficiently in [bmim][BF_4_] when the reaction was performed at 50 °C and in the presence of 50 equivalents of the corresponding nucleophile.

Evaluating the experimental results obtained for fluororotransformations of 1,1-diphenylethene (**15**) with F-TEDA-BF_4_ as fluorinating reagent in the presence of a nucleophile in ILs, in comparison with published results carried out in organic solvents (a mixture MeCN/MeOH, their ratio corresponding to 9/1) [31], preference should be given to the use of organic solvents. This can be ascribed to the fact that in a mixture of MeCN/MeOH (9/1 ratio) substrates of alkenic moiety were quantitatively converted into the corresponding *vicinal* methoxyfluoroalkanes, following Markovnikov type of regioselectivity at room temperature and for this transformastion required a considerably shorter reaction time, while authors do not report on the formation of side products [31]. On the other hand, for the formation of *vicinal* hydroxyfluoroakanes priority should be given to water [33] as reaction medium over ILs.

The investigation of fluorination with F-TEDA-BF_4_ in IL media was further expanded to methoxy substituted benzene derivatives. During previous studies in our laboratory, we already reported regulation of the regioselectivity of fluorination of aryl alkyl ketones with N-F reagents by the proper choice of organic solvent. While in acetonitrile fluorofunctionalization took place efficiently and regioselectively onto the aromatic ring, α-alkyl position next to the carbonyl group was selectively fluorinated when MeOH was used as reaction solvent [34]. In an attempt to fluorinate some aryl alkyl ketones (**19**, **20**, **21**, **22**) with F-TEDA-BF_4_ in ILs, we found that in addition to the substantially lower efficiency of fluorotransformations of these substrates in IL media also the aryl/alkyl selectivity failed to be controlled (Table 3).

**Table 3:** Transformations of aryl alkyl ketones with F-TEDA-BF_4_ in ILs^a^.

Entry	Substrate	Ionic liquid	t (h)	Conv.^b^	Products	Product distribution^c^
1	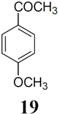	[bmim][BF_4_]	5	10%	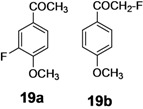	**19a**: 9%, **19**: 90%^d^
2			18	14%		**19a**: 11%, **19b**: 2%, **19**: 86%^d^
3		[bmim][PF_6_]	5	6%		**19a**: 6%, **19**: 94%
4			18	10%		**19a**: 9%, **19**: 90%^d^
5	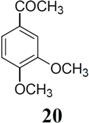	[bmim][BF_4_]	20	28%	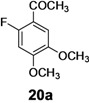	**20a**: 28%, **20**: 72%
6		[bmim][PF_6_]	20	7%		**20a**: 7%, **20**: 93%
7	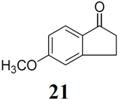	[bmim][BF_4_]	5	29%	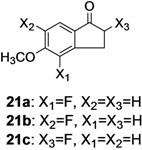	**21a**: 17%, **21b**: 5%, **21c**: 7%, **21**: 71%
8			18	47%		**21a**: 27%, **21b**: 7%, **21c**: 13%, **21**: 53%
9		[bmim][PF_6_]	5	18%		**21a**: 12%, **21b**: 3%, **21c**: 3%, **21**: 82%
10			18	10%		**21a**: 7%, **21b**: 2%, **21c**: 1%, **21**: 90%
11	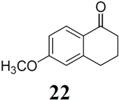	[bmim][BF_4_]	5	49%	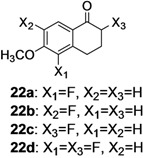	**22a**: 29%, **22b**: 6%, **22c**: 11%, **22d**: 3%, **22**: 51%
12			18	61%		**22a**: 30%, **22b**: 7%, **22c**: 16%, **22d**: 8%, **22**: 39%
13		[bmim][PF_6_]	5	22%		**22a**: 16%, **22b**: 3%, **22c**: 3%, **22**: 78%
14			18	11%		**22a**: 8%, **22b**: 2%, **22c**: 1%, **22**: 89%

^a^* Reaction conditions*: 0.2 mmol substrate, 0.24 mmol F-TEDA-BF_4_, 1 mL ionic liquid, 70 °C; ^b^ Conversion of substrate determined from ^1^H- and ^19^F-NMR spectra of the crude reaction mixture; ^c^ The distribution of compounds in the reaction mixture was determined from ^1^H- and ^19^F-NMR spectra; ^d^ Also the presence of 1% of 4-fluoroanisole.

The substituted benzaldehydes (**23**: 4-methoxybenzaldehyde, **24**: 3,4-(methylenedioxy)-benzaldehyde, and **25**: 4-hydroxybenzaldehyde) remained unchanged in the reactions with F-TEDA-BF_4_, performed in the investigated ILs. A slight transformation was noticed only in the case of the reaction of 4-methoxybenzaldehyde (**23**) with F-TEDA-BF_4_, carried out for 20 h at 70 °C in [bmim][BF_4_], where the substrate was converted into 3-fluoro-3-methoxybenzaldehyde (**23a**, 9%).

**Figure 3:**
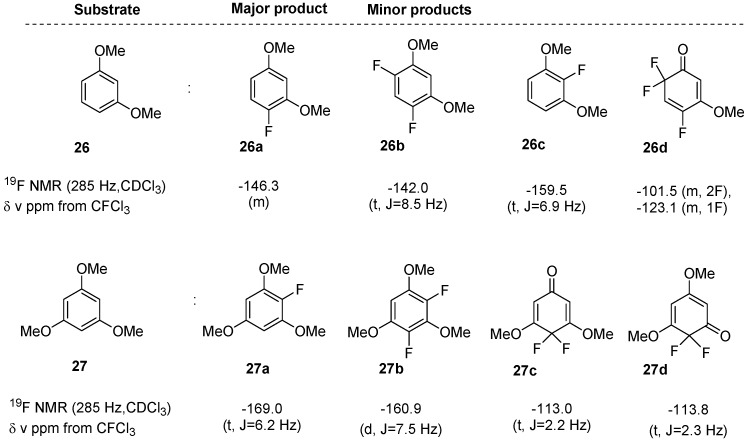
The mixture of products obtained in reaction of 1,3-dimethoxybenzene (**26**) or 1,3,5-trimethoxybenzene (**27**) with F-TEDA-BF_4_ in the IL [bmim][BF_4_] or [bmim][PF_6_] (70 °C, 3-4 h).

In the cases of dimethoxy- and trimethoxy-substituted benzenes, the fluorotransformation with F-TEDA-BF_4_ in IL media proved to be crucially dependent on the structure of the substrate. In the case of 1,3-dimethoxybenzene (**26**) and 1,3,5-trimethoxybenzene (**27**), which are both highly activated toward electrophilic substitution, the corresponding reaction mixture in [bmim][BF_4_], as well as in [bmim][PF_6_], contained at least 4 different products after 3 h reaction performed at 70 °C (Figure 3). On the other hand, the substrates 1,2-dimethoxybenzene (**28**), 1,4-dimethoxybenzene (**29**) and 1,2,3-trimethoxybenzene (**30**) remained mostly unreacted or fluoro-functionalization occurred only to a minor extent. 1,3-Dimethoxybenzene (**26**) in a reaction performed for 3 h at 70 °C in [bmim][BF_4_] was over 80% converted into a mixture of fluorinated products, with the formation of 4-fluoro-1,3-dimethoxybenzene (**26a**) as the major product, while from the ^19^F-NMR spectra the presence of the minor products 4,6-difluoro-1,3-dimethoxybenzene (**26b**), 2-fluoro-1,3-dimethoxybenzene (**26c**) and 4,6,6-trifluoro-3-methoxy-2,4-cyclohexadienone (**26c**) was established. The substrate **26** subjected to similar reaction conditions in [bmim][PF_6_] was 60% converted into a mixture of products **26a**, **26b**, **26c** and **26d**, with 4-fluoro-1,3-dimethoxybenzene (**26a**) prevalent. The efficiency of transformation and the distribution of the fluorinated products formed resembled the outcomes of reactions which occur under comparable reaction conditions in MeCN or in MeOH. The reaction of another reactive substrate, namely 1,3,5-trimethoxybenzene (**27**) with F-TEDA-BF_4_ in the studied IL media after 4 h at 70 °C provided a mixture of the products: 2-fluoro-1,3,5-trimethoxybenzene (**27a**, >50%) and in smaller quantities 2,4-difluoro-1,3,5-trimethoxybenzene (**27b**, ~5%), 4,4-difluoro-3,5-dimethoxy-2,5-cyclohexadienone (**27c**, 10-20%) and 2,2-difluoro-3,5-dimethoxy-3,5-cyclohexadienone (**27d**, <5%). Regarding the efficiency and the product distribution of the reaction, this was a similar result to the case when fluorination with F-TEDA-BF_4_ was carried out in MeCN [35] or in MeOH.

## 3. Experimental

### 3.1. General

The reactions were performed in 5-mL round bottom flasks with 1 mL of IL ([bmim][BF_4_] or [bmim][PF_6_]) as reaction medium. Firstly, F-TEDA-BF_4_ (0.24 mmol) was vigorously stirred in the IL at 70 °C until its complete dissolution. Then, substrate (0.2 mmol) was added and the reaction mixture continued to be stirred at the appropriate reaction temperature (22-70 °C). After the reaction had ceased, the products were extracted with *t*-BuOMe (4x2 mL), the combined organic phase was washed with water and after drying of the organic phase over anhydrous Na_2_SO_4_, the insoluble material was filtered off, while the reaction mixture was concentrated *in vacuo*. The crude reaction mixture was analyzed by TLC, ^1^H-NMR, ^19^F-NMR and MS and the products identified on the basis of comparison of their spectroscopic data with literature values [27,28,29,30,33,34,35,36,37,38,39,40,41,42,43,44,45,46,47,48,49,50,51].

## 4. Conclusions

The transformations of organic molecules with F-TEDA-BF_4_ investigated in the hydrophilic IL [bmim][BF_4_] and in the hydrophobic IL [bmim][PF_6_] and their evaluation in comparison to the corresponding reactions carried out in organic solvents revealed that these ILs are less convenient media for the fluoro-functionalization of alkyl substituted phenols (**4a**, **4b**, **4c**, **9**, **13**), 1,1-diphenyl- ethene (**15**) and methoxy-substituted aryl alkyl ketones (**19**, **20**, **21**, **22**) than MeCN or MeOH. Reactions of alkyl substituted phenols, namely 4-methylphenol (**4a**), 4-*i*-propylphenol (**4b**), 4-*t*-butyl- phenol (**4c**), 2,4,6-tri-*t*-butylphenol (**9**) and 3,4,5-trimethylphenol (**13**), with F-TEDA-BF_4_, depending on the substrate structure and reaction conditions, lead to the formation of either of the following fluorofunctionalised products: the corresponding *ortho*-fluorosubstituted product **5** as a result of electrophilic substitution at the *ortho* position of the phenol, difluorocyclohexadienone **6** formed following another fluorine introduction into **5**, 4-alkyl-4-fluoro-cyclohexa-2,5-dienone (**7**) as a consequence of the addition-elimination process after fluorine attack at the *para* position of 4-alkyl phenol, while 4-fluorophenol (**8**) was produced through *ipso* substitution and dealkylation at the *para* position. The transformations were higher in the hydrophilic IL [bmim][BF_4_] than in the hydrophobic [bmim][PF_6_]; however, compared to the published results of reactions performed in MeOH or in MeCN they were considerably less efficient and with a different distribution of products. Also for a substrate with an aromatic alkene moiety, namely 1,1-diphenylethene (**15**), when reacted with F-TEDA-BF_4_ a higher conversion was obtained in the hydrophilic IL than in the hydrophobic one. In addition, a markedly different selectivity of product formation was observed in the studied ILs. In [bmim][PF_6_], which with water forms a two-phase system, the substrate **15** was, regardless of the nucleophile present (MeOH, H_2_O or MeCN), transformed into an addition-elimination product, namely 1,1-diphenyl-2-fluoroethene (**17**), with high selectivity. On the other hand, when the reaction was performed in [bmim][BF_4_] the major product corresponded to *vicinal* hydroxy- or methoxy fluoroalkane (**16b** or **16a**). In an attempt to fluorofunctionalize some methoxy substituted aryl alkyl ketones using F-TEDA-BF_4_ in ILs, we found that in addition to the very low efficiency of the transformation, the reactions also proved unselective, thus giving preference to MeOH or MeCN for these fluoro-functionalizations. The efficiency of fluorination and the distribution of products in the cases of the reactions of 1,3-dimethoxybenzene (**26**) and 1,3,5-trimethoxybenzene (**27**) with F-TEDA-BF_4_ in the studied ILs was similar to the outcome of the reactions performed in MeCN or MeOH. Finally, we would like to point out that a small variation in reaction conditions may severely affect the outcome. Moreover, due to the lack of data on their physico-chemical properties, as well as long-term environmental influence of ILs one should be very cautious before proclaiming them as ‘greener’ alternative to organic solvents.
